# Neonatal pulmonary hypertension after severe early-onset fetal growth restriction: post hoc reflections on the Dutch STRIDER study

**DOI:** 10.1007/s00431-021-04355-x

**Published:** 2022-01-12

**Authors:** Anouk Pels, Wes Onland, Rolf M. F. Berger, Arno F. J. van Heijst, Enrico Lopriore, Irwin K. M. Reiss, Jacqueline Limpens, Sanne J. Gordijn, Wessel Ganzevoort

**Affiliations:** 1 Amsterdam UMC, Department of Obstetrics and Gynecology, University of Amsterdam, Meibergdreef 9, Amsterdam, The Netherlands; 2 Amsterdam UMC, Department of Neonatology, Emma Children’s Hospital, University of Amsterdam, Meibergdreef 9, Amsterdam, The Netherlands; 3 Department of Pediatric Cardiology, University Medical Center Groningen, Beatrix Children’s Hospital, University of Groningen, Groningen, The Netherlands; 4 Department of Neonatology, Radboud University Medical Center, Amalia Children’s Hospital, Nijmegen, The Netherlands; 5 Department of Neonatology, Leiden University Medical Center, Leiden, The Netherlands; 6 Department of Neonatology, Erasmus University Medical Center, Rotterdam, The Netherlands; 7 Amsterdam UMC, Medical Library, University of Amsterdam, Amsterdam, The Netherlands; 8 Department of Obstetrics and Gynecology, University Medical Center Groningen, University of Groningen, Groningen, the Netherlands

**Keywords:** Fetal growth restriction, Neonatal morbidity, Neonatal mortality, Pulmonary hypertension, Sildenafil

## Abstract

The aim was to reflect on the unexpected finding of persistent pulmonary hypertension of the neonate (PPHN) and pulmonary hypertension in infants born within the Dutch STRIDER trial, its definition and possible pathophysiological mechanisms. The trial randomly assigned pregnant women with severe early-onset fetal growth restriction to sildenafil 25 mg three times a day versus placebo. Sildenafil use did not reduce perinatal mortality and morbidity, but did result in a higher rate of neonatal pulmonary hypertension (PH). The current paper reflects on the used definition, prevalence, and possible pathophysiology of the data on pulmonary hypertension. Twenty infants were diagnosed with pulmonary hypertension (12% of 163 live born infants). Of these, 16 infants had PPHN shortly after birth, and four had pulmonary hypertension associated with sepsis or bronchopulmonary dysplasia. Four infants with PPHN in the early neonatal period subsequently developed pulmonary hypertension associated with bronchopulmonary dysplasia in later life. Infants with pulmonary hypertension were at lower gestational age at delivery, had a lower birth weight and a higher rate of neonatal co-morbidity. The infants in the sildenafil group showed a significant increase in pulmonary hypertension compared to the placebo group (relative risk 3.67; 95% confidence interval 1.28 to 10.51, *P* = 0.02).

*Conclusion:* Pulmonary hypertension occurred more frequent among infants of mothers allocated to antenatal sildenafil compared with placebo. A possible pathophysiological mechanism could be a “rebound” vasoconstriction after cessation of sildenafil. Additional studies and data are necessary to understand the mechanism of action.

**Table:** 

**What is Known:** *• In the Dutch STRIDER trial, persistent pulmonary hypertension in the neonate (PPHN) was more frequent among infants after antenatal sildenafil exposure versus placebo.*	
**What is New:** *• The current analysis focuses on the distinction between PPHN and pulmonary hypertension associated with sepsis or bronchopulmonary dysplasia and on timing of diagnosis and aims to identify the infants at risk for developing pulmonary hypertension.* *• The diagnosis pulmonary hypertension is complex, especially in infants born after severe early-onset fetal growth restriction. The research field could benefit from an unambiguous consensus definition and standardized screening in infants at risk is proposed.*

**Supplementary Information:**

The online version contains supplementary material available at 10.1007/s00431-021-04355-x.

## Introduction

Fetal growth restriction (FGR) is a condition in which the fetus does not reach its own intrinsic growth potential, most frequently caused by placental insufficiency [1]. In the extreme phenotype of early-onset FGR (before 32 weeks’ gestation), the predominant pathological pathway starts in early pregnancy when the necessary low-resistance high-flow placental bed circulation is inadequately established, resulting in a high-resistant placental circulation [2]. If born alive, usually through iatrogenic premature birth, the neonate is likely to be admitted to the neonatal intensive care unit (NICU) and is prone to neonatal mortality and morbidity [3].

In the Dutch STRIDER (Sildenafil TheRapy In Dismal prognosis Early onset fetal growth Restriction) trial [4], the phosphodiesterase 5 (PDE5) inhibitor sildenafil was evaluated as treatment for severe early-onset FGR in a randomized controlled setting. Sildenafil prevents formation of guanine monophosphate (GMP) out of cyclic GMP. This leads to an increased effect of nitric oxide, leading to vascular smooth muscle relaxation [5]. Previous studies suggested that sildenafil would improve uteroplacental flow, through which a higher gestational age and birth weight could be established. This led to the hypothesis that sildenafil would reduce the risk of perinatal mortality and morbidity. This hypothesis has been investigated in the Dutch STRIDER trial. No direct effect on the fetus was anticipated, but only through improved placental function. The primary outcome of the Dutch STRIDER trial was a composite of fetal and neonatal mortality or major neonatal morbidity.

The current randomized, placebo-controlled trial was terminated in July 2018 after the pre-planned interim analysis when the primary outcome was available for 50% (*n* = 216) of the planned sample size, based on advice of the data safety monitoring board (DSMB) who found potential harm for the infants in the face of futility. The concerns of harm were based on the increased incidence of pulmonary hypertension (PH) in infants whose mothers were allocated to sildenafil. The results are elsewhere described in detail [4]. No differences in the primary outcome or secondary outcomes were observed, and therefore, we concluded that sildenafil does not reduce the risk of perinatal morbidity and mortality.

After the trial was stopped, we aimed to validate, typify, and characterize the diagnosis of PH, and establish the association with neonatal deaths. The aim of this paper is to describe more detailed information on the infants diagnosed with PH, compare the characteristics and co-morbidities of this group to infants not diagnosed with PH, and to reflect on the pathophysiology of PH in this study group.

## Material and methods

Ethical approval for the Dutch STRIDER trial was given on July 22, 2014 (2014–131) by Amsterdam UMC. The trial was funded by the Netherlands Organization for Health Research and Development (ZonMW project no. 836021023). After discontinuation of the trial and because of the unexpected finding, the study team decided to install a blinded external adjudication committee of neonatologists (WO, AFJvH, EL, IKMR) and a pediatric cardiologist (RMFB) with expertise in PPHN/PH and preterm infants with FGR to review and study all potential cases of PH. In the study database, no distinction was made between PPHN and late-onset PH, associated with (developing) BPD and/or sepsis. Echocardiography was not performed in all infants as outcome in the trial, but based on local protocol of routine care, only on clinical indication at discretion of the treating physician. In the Netherlands, no standard echocardiography is performed in infants born after FGR or prematurity. Therefore, echocardiography is only performed if a suspicion of PPHN or other cardiac problems have been raised. This is in line with the other international STRIDER studies, in which also no routine echocardiography was performed in this population.

In accordance with criteria defined in consensus within the expert committee, a diagnosis of PPHN was applied when there was a clinical suspicion of PH, confirmed by either a more than 10% transcutaneous oxygen saturation difference between the upper right and lower limb (pre- and postductal differences) and/or right-left-shunting through an open foramen ovale and/or an open ductus arteriosus on echocardiography. A diagnosis and evaluation of severity of late-onset PH were made when PH was diagnosed after an initial period (roughly the first days of life) without signs of PH and based on echocardiographic criteria. In the Netherlands, echocardiographic diagnosis of pulmonary hypertension in newborns is performed according to international standards. At that time (2015), this was according to criteria proposed by Mourani et al., which included the following findings: any cardiac shunt with bidirectional or right-to-left flow an estimated right ventricular systolic pressure (RVSP) greater than 40 mm Hg, RVSP/systemic systolic blood pressure greater than 0.5, or any degree of ventricular septal wall flattening (all-in the absence of right ventricular outflow tract/pulmonary valve obstruction [6].

These criteria are more or less similar to those described in the POCUS guidelines for critically ill neonates by Singh et al., which were published not before finishing the STRIDER trial [7].

The clinical course and timing of diagnosis of the infants was discussed by all five experts, based on the charts and discharge letters. The discharge letters and, if necessary, additional notes from the patient charts, of all infants who received additional oxygen, were systematically reviewed. Four items were systematically discussed in order to come to a consensus diagnosis: the timing of occurrence of symptoms, the parameters on which the diagnosis PH was based (either cardiac ultrasound or saturation differences of more than 10%), the comorbidities (such as BPD, sepsis and lung hypoplasia), and, if applicable, the cause of death. When signs of PH were present within the first days of life, this was classified as PPHN. When the symptoms occurred after 28 days of life combined with an episode of sepsis, or with (developing) BPD [8–12], the diagnosis of late-onset PH was made. For all infants presenting with symptoms of PH between 48 h and 28 days, the course of symptoms and cardiorespiratory condition were reviewed and discussed. Consequently, an infant could be diagnosed with more than one type of PH, presenting initially with PPHN and later with late-onset PH. The cause of death was often multifactorial; the main course of death, as highlighted in the discharge letter or charts, was identified and recorded. If necessary, more information on the clinical course and/or results of diagnostic tests was asked for at the local neonatologist. The timing of PPHN was described in days after delivery, considering the day of delivery as day 0. The data on the diagnosis PH were linked to other clinical data collected in the study database, such as gestational age at delivery, birth weight, maternal hypertensive disorder, antenatal Doppler measurements, and neonatal comorbidities. Given the small number of infants, formal statistical testing was not performed.

## Results

The interim analysis and the termination of the Dutch STRIDER trial took place in July 2018, after the primary outcome was collected for 50% of the planned sample size. At that time, 216 (60% of the predetermined population) pregnant women had been randomized in the trial, 20 of whom were still pregnant or the infant was still hospitalized at the NICU at the time of the interim analysis. One participant was lost to follow-up for all maternal and fetal/neonatal outcomes due to moving abroad during the pregnancy (Supplemental Fig. 1). Additional information on inclusion criteria and baseline characteristics has been described elsewhere [4].

In total, of the 85 infants allocated to sildenafil, the expert committee found that 16 (19%) experienced PH (either PPHN or late-onset PH or both) whereas this was the case for four (5%) of the 78 infants in the placebo group (risk ratio (RR) 3.67; 95% confidence interval (CI) 1.28 to 10.51; *p* = 0.02) (Table 1). Of the 16 infants with PH in the sildenafil group, ten died (63%). In the placebo group, three out of four infants died (75%) (RR 0.83; 95% CI 0.42 to 1.65; *p* = 0.60). Of the 16 infants that were diagnosed with PPHN (13 allocated to sildenafil and three to placebo), four also developed late-onset PH associated with (developing) BPD (three allocated to sildenafil and one to placebo). Two infants allocated to sildenafil experienced late-onset PH associated with sepsis (both allocated to sildenafil), whereas two infants experienced late-onset PH associated with (developing) BPD (one allocated to sildenafil, one to placebo) (Table 1).

**Table 1:** Types of pulmonary hypertension within live born infants in the Dutch STRIDER trial, per randomization allocation group

	Sildenafil (*n* = 85)		Placebo (*N* = 78)	
	Neonatal death (*n* = 21)	Survival to discharge (*n* = 64)	Neonatal death (*n* = 11)	Survival to discharge (*n* = 67)
Total pulmonary hypertension	10	6	3	1
PPHN	7	3	1	1
PH associated with sepsis	1	1	0	0
Late-onset PH associated with (developing) BPD	1	0	1	0
PPHN followed by late-onset PH associated with (developing) BPD	1	2	1	0

The total number of infants in the sildenafil group with any PH compared with the placebo group was 16/85 (19%) versus 4/78 (5%); RR 3.67; 95% CI 1.28 to 10.51; *P* = 0.008

*PPHN* persistent pulmonary hypertension of the neonate, *PH* pulmonary hypertension, *BPD* bronchopulmonary dysplasia

Table 2 presents the characteristics of the infants diagnosed with PH (either PPHN or late-onset PH), compared with the infants without PH. The median gestational age at delivery and birth weight were lower in the infants that experienced PH. A higher percentage of the infants that experienced PH had absent or reversed EDF in the umbilical artery at randomization. Furthermore, a shorter treatment period was observed in the infants that were diagnosed with PH. Relevant neonatal morbidities (moderate or severe BPD, ROP, and sepsis) as well as the primary outcome (fetal or neonatal mortality or survival with major neonatal morbidity) occurred more often in the group infants experiencing PH. For purpose of comparison, we presented the data of the subgroup infants with PPHN alongside data of the infants experiencing any late-onset PH. No relevant differences were observed.

**Table 2:** Characteristics of infants with and without any pulmonary hypertension and infants with PPHN

	Infants with PPHN (*n* = 16)	Infants with late-onset PH only (*n* = 4)	Infants without PH (*n* = 143)
GA at randomization (weeks) (median, IQR)	24.9 (22.2 to 25.4)	25.1 (23.6 to 26.8)	25.4 (24.0 to 26.6)
EFW at randomization (g) (median, IQR)	467 (283.0 to 529.0)	530.5 (441.5 to 672)	518 (375.0 to 646.0)
Absent or reversed EDF in the umbilical artery at randomization (%)	6 (37.5%)	3 (75.0%)	36 (25.2%)
Female sex (%)	6 (37.5%)	1 (25.0%)	71 (49.7%)
Maternal pre-eclampsia or HELLP (%)	8 (50.0%)	3 (75.0%)	66 (46.2%)
Days of treatment study medication (median, IQR)	11 (7.0 to 28.0)	14.0 (9.3 to 21.0)	24.5 (9.8 to 42.0)
GA at delivery (weeks) (median, IQR)	26.6 (26.0 to 27.8)	28.3 (28.0 to 28.7)	29.6 (27.9 to 34.1)
Incomplete course corticosteroids (< 48 h before delivery) (%)	2 (12.5%)	0 (0%)	9 (6.3%)
Complete course corticosteroids (48 h to 14 days before delivery) (%)	10 (62.5%)	2 (50.0%)	96 (67.1%)
Birthweight (g) (median, IQR)	522.5 (432.5 to 611.3)	740.0 (620.0 to 815.0)	805.0 (638.0 to 1470.0)
Primary outcome (%)	16 (100%)	3 (75.0%)	52 (36.4%)
Neonatal mortality (%)	10 (62.5%)	3 (75.0%)	19 (13.3%)
IVH grade 3 or more (%)	1 (6.3%)	0 (0%)	4 (2.8%)
Moderate or severe BPD (%)	8 (50.0%)	2 (50.0%)	29 (20.3%)
ROP treated by laser or surgery (%)	5 (31.3%)	0 (0%)	6 (4.2%)
One or more culture-proven or clinical suspected infection (%)	13 (81.3%)	4 (100%)	62 (43.4%)
NEC grade 2 or more (%)	2 (12.5%)	1 (25.0%)	12 (8.4%)

Primary outcome = fetal or neonatal mortality or survival with major neonatal morbidity, defined as IVH grade 3 or more, PVL grade 2 or more, moderate or severe BPD, NEC grade 2 or more or ROP requiring surgery

*PH* pulmonary hypertension, *GA* gestational age, *IQR* inter quartile range, *EFW* estimated fetal weight; *EDF* end-diastolic flow, *HELLP* Haemolysis Elevated Liver enzymes Low Platelets

**Fig. 1:**
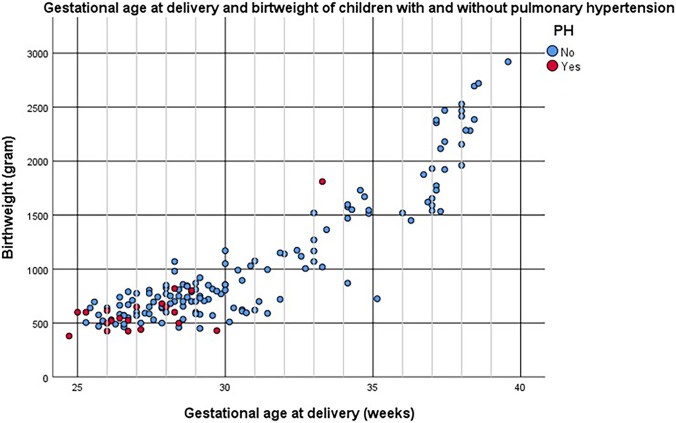
Gestational age at delivery and birth weight of infants with and without PH

Figure 1 presents the gestational age and birth weight of the infants diagnosed with PH and the infants not diagnosed with PH. Almost all infants were below 30 weeks gestational age at delivery and had a birth weight below 1000 g. When reviewing the association between the degree of FGR and PH, no association was found in this extreme subpopulation between the degree of FGR, as assessed by the standard deviation below the mean birth weight for gestational age, and the risk of PH (Supplemental Fig. 2).

Of the twenty infants with PH, data on respiratory support have been collected. Median number of days of supplemental oxygen was 58.0 days (IQR 3.0 to 85.0 days). Seven infants received postnatal steroids for preventing BPD. Nineteen infants were diagnosed with respiratory distress syndrome (RDS), of who 18 received one or more instillations surfactant. One infant with lung hypoplasia received surfactant as well, without diagnosis of RDS. Mean number of surfactant instillations was 2.6 (three infants received four instillations, seven infants three instillations, eight infants two instillations, and one infant one instillation).

Regarding the treatment of PH, data on the use of inhaled nitric oxide (iNO) have been collected. Eighteen out of 20 infants with PH were treated with iNO. No details on the treatment protocol and dosage were collected. The discharge letters of the infants with PH were reviewed in order to find information on the NO response. Of 11 infants with available information on NO response, two infants responded to NO and nine did not. Of the other six infants who had PH and received NO, no information on NO response was available. From these data, it seems most infants were non-responders for NO. Even though the trial was not powered for this outcome, this could support the hypothesis that antenatal sildenafil use might influence the sensitivity for postnatal NO treatment. We could not identify other studies on this topic. Further studies are needed to investigate this hypothesis.

When looking at pulmonary comorbidity, of the 16 infants with PPHN, no pulmonary hemorrhage was reported, one infant was diagnosed with pneumothorax, and two infants had pulmonary interstitial emphysema. One infant had an umbilical arterial pH below 7.00 (of eight infants, the umbilical artery pH was missing) and six infants had an Apgar score below 7 at 5 min. One infant had culture-proven early-onset sepsis (defined as < 72 h after birth). The outcome lung hypoplasia was not systematically collected as outcome, but of the 16 infants with PPHN, the discharge letters were reviewed, and one infant was diagnosed with lung hypoplasia, most likely based on an anhydramion (due to congenital nephropathy). The discharge letters of the other infants do not mention lung hypoplasia.

**Fig. 2:**
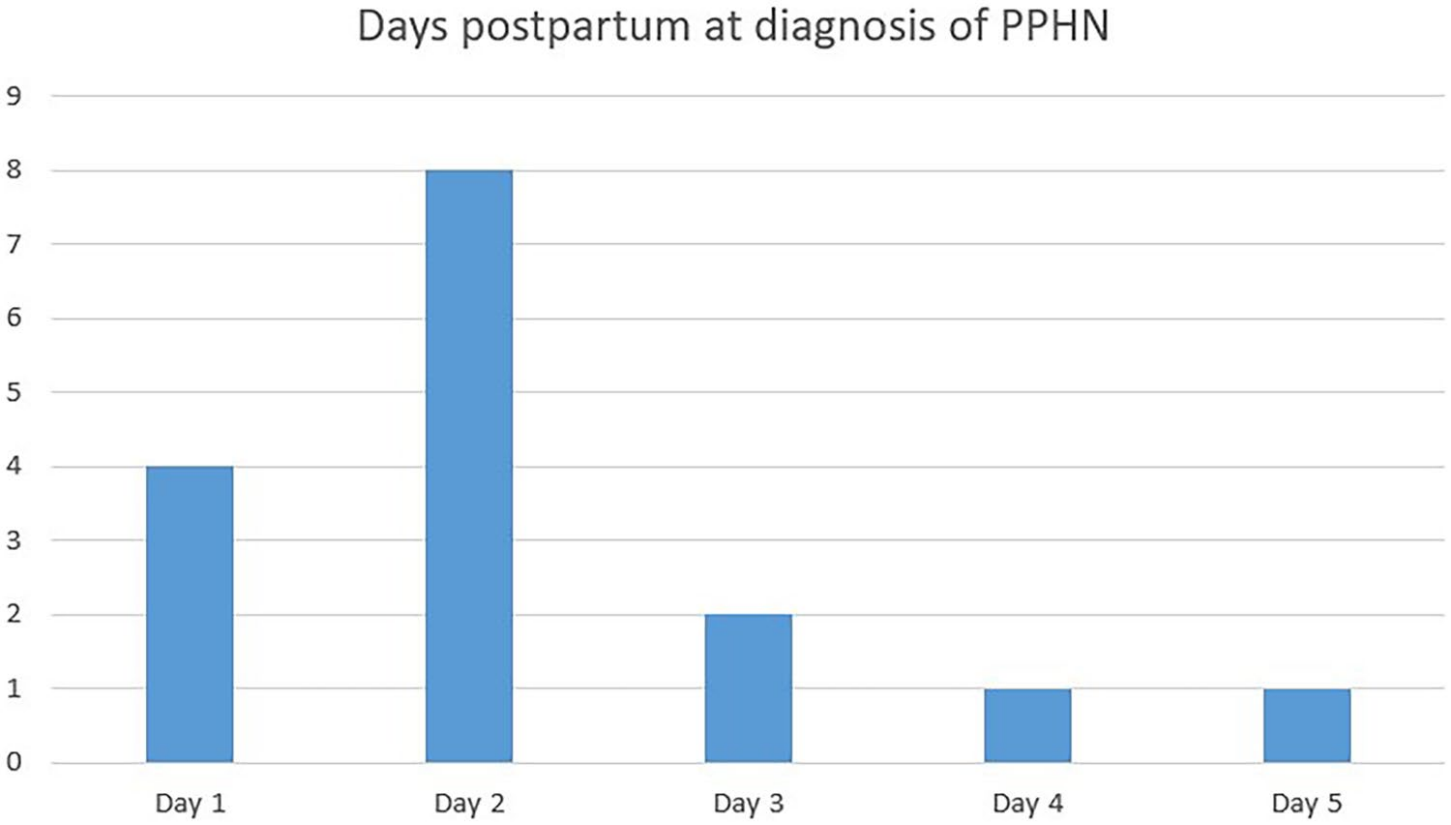
Timing of diagnosis of PPHN

The timing of PPHN is presented in Fig. 2. Most infants were diagnosed on day 1 or 2. All infants received the diagnosis within the first 5 days (the day of delivery was considered as day zero). PH associated with sepsis occurred in two infants: on day 2 and day 5. The occurrence of PH associated with (developing) BPD was widespread: on days 20, 61, 85, 88, 103, and 106.

When reviewing the cause of death, PH was determined to be the primary cause of death in four infants: two allocated to sildenafil and two to placebo. Ages of death were 3, 4, 6, and 308 days postpartum respectively; first three cases were diagnosed with PPHN, and the last case was diagnosed first with PPHN and later on with PH associated with BPD. Whether or not these infants were treated with rescue vasodilators or not was not collected systematically. However, the national Dutch guideline prescribes treatment with vasopressors as norepinephrine and vasodilators such as milrinone [13].

## Discussion

The Dutch STRIDER study revealed an unexpected higher incidence of PH (PPHN or late-onset PH) in growth-restricted infants antenatally exposed to sildenafil, compared with placebo. Despite the fact that this post hoc analysis was not pre-announced in the study protocol, it was deemed important to explore this unexpected finding. After studying the data on PH, twenty out of 163 (12%) of live born infants in the Dutch STRIDER trial were diagnosed with PH of whom 16 (75%) received sildenafil and four (25%) placebo. The majority of the infants experienced PPHN during the first days after delivery, but also cases of late-onset PH associated with (developing) BPD and PH associated with sepsis were observed. PPHN occurs in about two out of 1000 live-born infants [14–16], but recent studies report an increased prevalence in preterm born infants [17, 18].

The current manuscript states that the definition, detection, and characterization of neonatal PH are complex and requires standardized screening in risk groups, such as preterm infants born after FGR. In the current subpopulation of the Dutch STRIDER study, the gestational age at delivery and birth weight were lower and the rate of neonatal morbidity higher, compared with infants without PH (in this small group, the morbidities moderate or severe BPD, ROP, and sepsis were significantly more common), representing the most vulnerable group. Future research is needed to investigate whether the risk of developing PH is associated with the (antenatally diagnosed) severity of FGR, or the absolute birth weight and gestational age at delivery are the most important risk factors in themselves.

The pathophysiological mechanism underlying the higher rate of PH in infants of whom the mother was allocated to sildenafil, compared to placebo, remains unclear. A “rebound” vasoconstriction (or lack of further vasodilation) was hypothesized to be a possible mechanism. Even though there is no evidence to support structural changes to the pulmonary vasculature, this cannot be ruled out as possible mechanism. In the small population of this sub study with the available level of detail, it was not possible to further elaborate on hypothesis or provide proof for either of them. However, the infants who were diagnosed with PH responded poorly to treatment with iNO (nine out of 11 with known response to iNO). Despite the low number of cases, this supports the hypothesis of rebound vasoconstriction with decreased sensitivity to iNO.

Only 13 autopsies have been performed among the infants that were stillborn (*n* = 9) or died in the neonatal period (*n* = 4). None of the infants with PH who subsequently died had an autopsy. In these autopsies, no pulmonary capillary density or other pulmonary abnormalities have been described. It would be interesting to investigate the lung capillaries in more detail in future autopsies in a comparable patient group. Unfortunately, we did not have data on levels of sildenafil in the fetal circulation or umbilical cord, which can be an interesting topic for future studies evaluating the effect of antenatal medication in order to be able to specify the association between the intervention and clinical findings. A recent ex vivo perfusion study of placenta tissue of healthy women and women with PE showed a decrease of baseline vascular tension after treatment with sildenafil and an absence of PDE5 upregulation in the placentas of women with PE, which is in line with the futility found in the current study [19]. The proposed rebound mechanism in which the pulmonary vasculature in utero was under the effect of sildenafil resulting in vasodilatation has face value. After the sudden cessation of sildenafil, one could imagine that the pulmonary vasculature fails in providing the necessary vasodilatation for extra-uterine life.

One of the limiting factors in our study was the lack of standardized echocardiography in infants at risk of PH and the lack of an international accepted definition of PPHN. We defined PPHN on forehand based on clinical experience as observing a difference in oxygen saturation of more than 10% between the right upper limb and the left upper or lower limb or confirmed by ultrasound. Of the 16 infants with PPHN, nine infants had a cardiac ultrasound that confirmed signs of PH. In seven infants, no cardiac ultrasound was performed since the diagnosis PPHN was based on differences in oxygen saturation and/or since the cardiac ultrasound would not change the course of action, and/or the clinical situation of the infant was too instable to perform a cardiac ultrasound. Thus, in seven infants, the diagnosis was made based on clinical variables only, and in nine infants, the diagnosis was made based on clinical variables, followed by cardiac ultrasound. A systematic screening of all infants in both groups at similar time points would have been a prerequisite for a valid comparison of the two groups. Furthermore, other potential useful diagnostics, such as lung X-rays and lung ultrasounds, have not been performed and collected systematically. Especially the role of lung ultrasound in this patient group would have been interesting, since this might be a promising technique in diagnosing PPHN and provide insight in the underlying cause [20–22].

Retrospective diagnosis was further complicated due to heterogeneity in neonatal clinical course. Some clinicians and researchers hypothesize that signs of PPHN typically start within the first days of life based on the idea that fetal circulation should adapt to extra-uterine life within the first day(s) [23]. However, others hypothesize that PPHN occurs throughout the neonatal period (first 28 days of life) [24]. We considered the timing of occurrence of symptoms important in the distinction between PPHN and late-onset PH, next to the existence of an underlying disease. There is scant data on how much time is “physiologically” appropriate to adapt from intrauterine toward neonatal life. We recognize that diagnoses of PPHN and late-onset PH can form a continuum, representing evolving pulmonary vascular disease due to disturbed vascular and airway development already in utero combined with environmental injuries [25]. The distinction between primary and secondary problems is not straightforward, especially in fragile infants, underlining the need for a (consensus) definition for clinical practice and research. Further studies are necessary to provide insight in the pathophysiological mechanisms in order to find an international accepted (consensus) definition. Also, further studies should focus on therapeutic options, especially for these vulnerable patients [26, 27]. Recently, a proposal for a therapeutic algorithm for treating life-threatening PPHN has been published. Since we observe a high rate of non-responding to iNO, of infants whose mother was treated with sildenafil in pregnancy, the response to iNO should be recorded as well in future studies.

The Dutch STRIDER study was part of an international collaboration, in which four similar trials with different focus were independently funded and executed [28]. The four studies prospectively investigated the same intervention, but reported a different primary outcome and included a different sample size, due to feasibility. The trials will contribute to a prospectively planned systematic review including individual patient data. The overarching hypothesis of this collaboration of clinical trials is that sildenafil compared with placebo will improve fetal growth and wellbeing, allowing prolongation of pregnancy leading to a decrease in the rate of fetal and neonatal mortality and severe morbidity [29].

The incidence of PH in the Dutch STRIDER trial (12%) is comparable to the UK STRIDER trial [30] (16.3%; 15 out of 92 infants, six allocated to sildenafil and nine to placebo, personal communication), but higher than in the NZAUS STRIDER trial [31] (1.9%; two out of 103 infants, one allocated to sildenafil and one to placebo). These differences may be explained by the minor differences in the baseline characteristics of participants, definition differences, diagnostic strategies, or thresholds of suspicion. In the UK and NZAUS STRIDER studies, also no routine screening for PPHN was performed. Since the infants with PH were spread equally across participating centers within our country, we consider it unlikely that clinical vigilance explains the difference between randomization groups. Additional studies and individual data (IPD) meta-analysis including the other international STRIDER trials will provide additional data [28].

The studies we identified in literature report a prevalence of PH among infants born after FGR between 5 and 33% [19–22]. Even though there are only few studies reporting the prevalence of PH among infants born after FGR, the percentage of 12.2% that we found does not seem to be an outlier. However, a prevalence of 5.1% (4 out of 78 infants) of PH was seen in our placebo group, which corresponds to the lowest percentage, reported in the study of Turan [32]. The included studies used different definitions of PH, including presence of right-to-left or bidirectional shunting, tricuspid regurgitation, flattening of interventricular septum, right ventricular hypertrophy, elevated right ventricular pressures, and abnormal pulmonary artery Doppler.

In conclusion, we report a prevalence of 12% PH among a group infants born after severe, early-onset FGR, in which almost all infants were born below 30 weeks of gestation and had a birth weight below 1000 g. A higher rate of neonatal (co-)morbidity was observed. In the group infants of whom the mother was treated with sildenafil, compared with placebo, a higher rate of PH was observed, for which a rebound vasoconstriction might be possible explanations. There is need for an international accepted (consensus) definition of PPHN and we propose standardized screening for PPHN in infants born after early-onset FGR born below 30 weeks of gestation and a birth weight below 1000 g and below 3rd percentile by echocardiography.

## The Dutch STRIDER Trial Group

M.A. de Boer, Department of Obstetrics and Gynecology, Amsterdam UMC, Vrije Universiteit Amsterdam, Amsterdam, the Netherlands. W.P. de Boode, Department of Neonatology, Radboud University Medical Center, Radboud Institute for Health Sciences, Amalia Children’s Hospital, Nijmegen, The Netherlands. J. Derks, Department of Obstetrics, Wilhelmina Children’s Hospital University Medical Center, Gynecology and Neonatology, Utrecht, the Netherlands. J. van Drongelen, Department of Obstetrics and Gynecology, Radboud University Medical Center, Nijmegen, The Netherlands. R.G. Duijnhoven, Department of Obstetrics and Gynecology, Amsterdam UMC, University of Amsterdam, Amsterdam, the Netherlands. J.J. Duvekot, Department of Obstetrics and Gynecology, Erasmus University Medical Center, Rotterdam, the Netherlands. A. Elvan-Taspinar, Department of Obstetrics and Gynecology, University Medical Center Groningen, Groningen, the Netherlands. J. van Eyck, Department of Obstetrics and Gynecology, Isala Hospital, Zwolle, the Netherlands. C. Gluud, The Copenhagen Trial Unit, Centre for Clinical Intervention Research, Rigshospitalet, Copenhagen University Hospital, Copenhagen, Denmark. H. van Goor, Department of Pathology and Medical Biology, section Pathology, University Medical Center Groningen, University of Groningen, Groningen, The Netherlands. J.C. Jakobsen, The Copenhagen Trial Unit, Centre for Clinical Intervention Research, Rigshospitalet, Copenhagen University Hospital, Copenhagen, Denmark; Department of Cardiology, Holbæk Hospital, Denmark; Department of Regional Health Research, The Faculty of Health Sciences, University of Southern Denmark, Denmark. E.M.W. Kooi, Division of Neonatology, Beatrix Children’s Hospital, University Medical Center Groningen, University of Groningen, Groningen, the Netherlands. J. van Laar, Department of Obstetrics and Gynecology, Maxima Medical Center, Veldhoven, the Netherlands. A.T. Lely, Department of Obstetrics, Wilhelmina Children’s Hospital University Medical Center, Gynecology and Neonatology, Utrecht, the Netherlands. P. Lemmers, Department of Neonatology, Wilhelmina Children’s Hospital, University Medical Center Utrecht, Utrecht, the Netherlands. Ben W. Mol, Department of Obstetrics and Gynecology, Monash University, Monash Medical Centre, Clayton, Australia. S.M. Mulder- de Tollenaer, Department of Pediatrics, Isala Hospital, Zwolle, the Netherlands. C.A. Naaktgeboren, Department of Obstetrics and Gynecology, Amsterdam UMC, University of Amsterdam, Amsterdam, the Netherlands. H.J. Niemarkt, Department of Neonatology, Maxima Medisch Centrum, Veldhoven, The Netherlands. S. Al-Nasiry, Department of Obstetrics and Gynecology, Maastricht University Medical Center, Maastricht, the Netherlands. L.P. Morssink, Department of Obstetrics and Gynecology, Medical Center Leeuwarden, Leeuwarden, the Netherlands. S.H.P. Simons, Department of Pediatrics, Division of Neonatology, Erasmus UMC Rotterdam, Sophia Children’s hospital, the Netherlands. M. Sueters, Department of Obstetrics and Gynecology, Leiden University Medical Center, Leiden, the Netherlands. K. Taghavi, Department of Obstetrics and Gynecology, Inselspital Bern, Frauenklinik, Bern, Switzerland. A.G. van Wassenaer-Leemhuis, Department of Neonatology, Emma Children’s Hospital, Amsterdam UMC, University of Amsterdam, Amsterdam, the Netherlands. M.M. van Weissenbruch, Department Pediatrics / IC Neonatology, Amsterdam UMC, Location VUmc, Amsterdam, The Netherlands.

## Supplementary Information

Below is the link to the electronic supplementary material.

Supplementary file1 (JPG 62 KB)

Supplementary file2 (JPG 71 KB)

## Data Availability

Upon request of the author.

## Abbreviations

BPD: Bronchopulmonary dysplasia
CI: Confidence interval
DSMB: Data safety monitoring board
FGR: Fetal growth restriction
GMP: Guanosine monophosphate
IVH: Intraventricular hemorrhage
LV: Left ventricle
NEC: Necrotizing enterocolitis
NICU: Neonatal intensive care unit
PAP: Pulmonary artery pressure
PDE5: Phosphodiesterase 5
PE: Preeclampsia
PH: Pulmonary hypertension
PPHN: Persistent pulmonary hypertension of the neonate
PVL: Periventricular leukomalacia
ROP: Retinopathy of prematurity
RV: Right ventricle
RR: Relative risk
STRIDER: Sildenafil therapy in dismal prognosis early-onset fetal growth restriction

## Code availability

N/A.
