# Supplementary figures and images for: Neonatal pulmonary hypertension after severe early-onset fetal growth restriction: post hoc reflections on the Dutch STRIDER study
Source: Eur J Pediatr. 2022 Jan 12;181(4):1709–18. doi: 10.1007/s00431-021-04355-x (PMC8964651; doi:10.1007/s00431-021-04355-x)

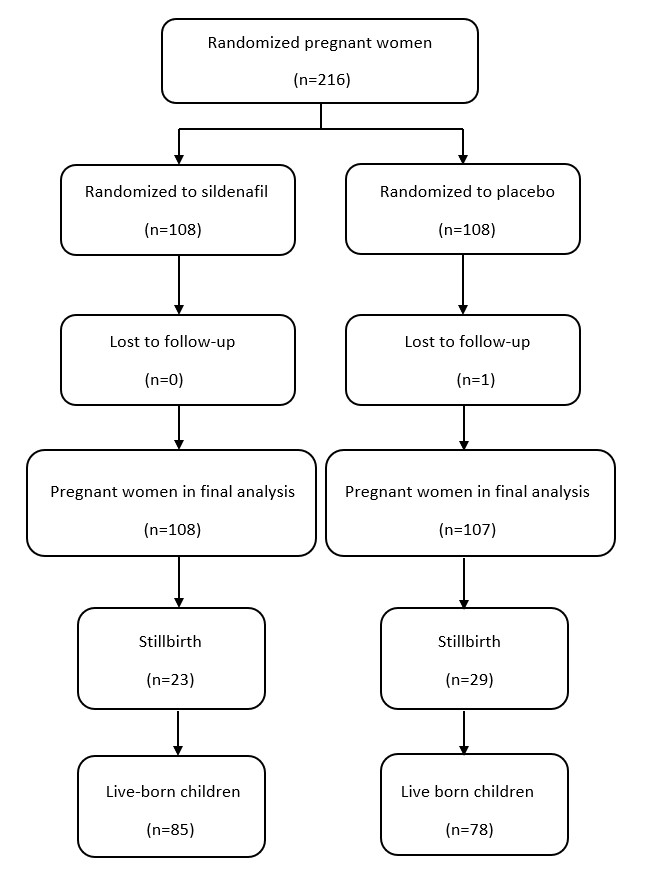

Supplement: Supplementary file 1 — Supplementary file1 (JPG 62 KB) [file 431_2021_4355_MOESM1_ESM.jpg]

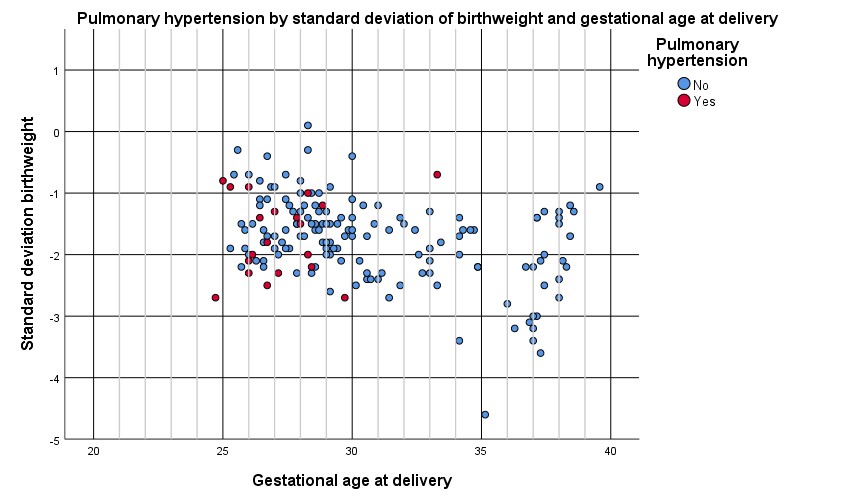

Supplement: Supplementary file 2 — Supplementary file2 (JPG 71 KB) [file 431_2021_4355_MOESM2_ESM.jpg]
